# Dynamics and Concordance Abnormalities Among Indices of Intrinsic Brain Activity in Individuals With Subjective Cognitive Decline: A Temporal Dynamics Resting-State Functional Magnetic Resonance Imaging Analysis

**DOI:** 10.3389/fnagi.2020.584863

**Published:** 2021-01-25

**Authors:** Yiwen Yang, Xinyi Zha, Xiaodong Zhang, Jun Ke, Su Hu, Ximing Wang, Yunyan Su, Chunhong Hu

**Affiliations:** ^1^Department of Radiology, The First Affiliated Hospital of Soochow University, Suzhou, China; ^2^Institute of Medical Imaging, Soochow University, Suzhou, China; ^3^Department of Radiology, Tianjin First Central Hospital, Tianjin, China

**Keywords:** subjective cognitive decline, Alzheimer's disease, resting-state functional MRI, temporal dynamics analysis, intrinsic brain activity

## Abstract

Individuals with subjective cognitive decline (SCD) are more likely to develop into Alzheimer disease (AD) in the future. Resting-state functional magnetic resonance imaging (rs-fMRI) studies have shown alterations of intrinsic brain activity (IBA) in SCD individuals. However, rs-fMRI studies to date have mainly focused on static characteristics of IBA, with few studies reporting dynamics- and concordance-related changes in IBA indices in SCD individuals. To investigate these aberrant changes, a temporal dynamic analysis of rs-fMRI data was conducted on 94 SCD individuals (71.07 ± 6.18 years, 60 female), 75 (74.36 ± 8.42 years, 35 female) mild cognitive impairment (MCI) patients, and 82 age-, gender-, and education-matched controls (NCs; 73.88 ± 7.40 years, 49 female) from the Alzheimer's Disease Neuroimaging Initiative database. The dynamics and concordance of the rs-fMRI indices were calculated. The results showed that SCD individuals had a lower amplitude of low-frequency fluctuations dynamics in bilateral hippocampus (HP)/parahippocampal gyrus (PHG)/fusiform gyrus (FG) and bilateral cerebellum, a lower fractional amplitude of low-frequency fluctuation dynamics in bilateral precuneus (PreCu) and paracentral lobule, and a lower regional homogeneity dynamics in bilateral cerebellum, vermis, and left FG compared with the other two groups, whereas those in MCI patients were higher (Gaussian random field–corrected, voxel-level *P* < 0.001, cluster-level *P* < 0.05). Furthermore, SCD individuals had higher concordance in bilateral HP/PHG/FG, temporal lobe, and left midcingulate cortex than NCs, but those in MCI were lower than those in NCs. No correlation between concordance values and neuropsychological scale scores was found. SCD individuals showed both dynamics and concordance-related alterations in IBA, which indicates a compensatory mechanism in SCD individuals. Temporal dynamics analysis offers a novel approach to capturing brain alterations in individuals with SCD.

## Introduction

The neurodegenerative changes that eventually develop into dementia due to Alzheimer disease (AD) begin to accumulate approximately 20 years before clinical symptoms appear (Sperling et al., 2011). Subjective cognitive decline (SCD) refers to the subjective experience of cognitive decline without any objective impairments detectable by cognitive assessments, and they are more likely to develop into AD in the future (Jessen et al., 2014; Rabin et al., 2017). The early diagnosis of AD-related SCD individuals is particularly important for AD prevention and intervention in clinical settings (Lin et al., 2019). In recent years, neuroimaging biomarkers capable of detecting the early neurodegenerative changes and dynamic alterations in neurological disorders on the AD spectrum have been identified, including SCD duration (Yang et al., 2018). A previous structural magnetic resonance imaging (sMRI) study demonstrated that a thinner cortical layer, particularly in the temporal cortex, is associated with steeper memory decline in SCD individuals (Verfaillie et al., 2018). Other researchers have also reported that SCD individuals have a greater white matter (WM) hyperintensity volume (van Rooden et al., 2018) and more severe WM fiber tract injury than controls (Brueggen et al., 2019). Moreover, SCD individuals exhibit higher amplitude of low-frequency fluctuations (ALFF) values in some brain regions compared with controls (Sun et al., 2016). Additionally, SCD individuals show alterations in their whole-brain functional connectivity strength (FCS), with the relative FCS found to be higher in posterior cingulate cortex (PCC)/precuneus (PreCu) (Dong et al., 2018). Furthermore, Rodda et al. found that while executing a divided attention task, SCD individuals showed increased activation in some brain regions compared to controls (Rodda et al., 2011). Li et al. assessed the intrinsic connectivity network of SCD individuals and found that SCD individuals show higher degree centrality (DC) in bilateral hippocampus (HP) and left fusiform gyrus (FG), but lower DC in the inferior parietal region than controls (Li et al., 2018). All these studies indicate a possible compensatory mechanism affecting the intrinsic brain activity (IBA) of SCD individuals. In addition, research has revealed no significant differences in cortical thickness in SCD individuals compared with healthy people (Sun et al., 2016), suggesting that the change in IBA might be a more sensitive biomarker for SCD than structure alterations.

To date, resting state functional MRI (rs-fMRI) studies have mostly focused on static characteristics, which involve calculations based on all time points during the scan as a whole. However, human brain activity is context-sensitive and activity-dependent, meaning that IBA is dynamic and fluctuates over time; this underlies functional integration in the brain (Park et al., 2018). Recently, studies of the temporal dynamics of IBA using analyses such as the sliding window method have yielded some important results. For instance, Mao et al. found gender differences in dynamic functional connectivity in healthy individuals (Mao et al., 2017). Similarly, dynamic alterations have also been reported in patients with depression (Kaiser et al., 2016; Li et al., 2019b; Wu et al., 2019), epilepsy (Wang et al., 2019), and Parkinson disease (Fiorenzato et al., 2019; Zhang et al., 2019). In addition, Yan et al. found that different rs-fMRI indices of IBA tend to exhibit a relatively high degree of concordance within and between individuals; this difference in concordance appears to be stable and is negatively related to age when taken as an interindividual measurement (Yan et al., 2017). However, to our knowledge, few studies to date have analyzed changes in the dynamics and concordance of IBA indices in SCD individuals.

To investigate changes in the dynamics of IBA in SCD individuals, we conducted a temporal dynamics analysis based on rs-fMRI data obtained from SCD individuals, and compared these with data from mild cognitive impairment (MCI) patients and controls (NCs), respectively. We hypothesized that there would be some differences in the dynamics and concordance of IBA among the three groups and that the alterations in SCD individuals would be intermediate between those in MCI patients and NCs.

## Materials and Methods

### Alzheimer's Disease Neuroimaging Initiative

Data used in this study were obtained from the Alzheimer's Disease Neuroimaging Initiative (ADNI) database (http://adni.loni.usc.edu). ADNI was launched in 2003 as a public–private partnership, led by principal investigator Michael W. Weiner, MD. The primary goal of ADNI is to test whether data from clinical and neuropsychological assessments, serial MRI, positron emission tomography, and other biological markers can be combined to measure the progression of AD.

### Participants

The research plan was approved by the review committee at each institution participating in ADNI. All subjects fully understood the research aims and signed an informed consent form. Individuals with self-report significant memory concern are recruited into significant memory concern cohort in ADNI. In the current study, this part of the population was included as the SCD group. SCD, MCI, and NC subjects who underwent structural, rs-fMRI scans on 3.0 T MRI (Siemens, Germany), in addition to receiving neuropsychological assessments in the same visit, were included (see Supplementary Material 1 for the specific inclusion criteria for subjects). Exclusion criteria were as follows: incomplete image or neuropsychological assessment data, severe neuropsychiatric disease, cerebral organic disease, history of brain injury or other persistent neurological diseases, or known structural abnormalities of the brain. According to the criteria above, a total of 291 were collected in this study; 40 individuals were excluded because of excessive head motion. Finally, 94 SCD individuals, 75 MCI patients, and 82 well-matched NCs were included in this study.

### Neuropsychological and MRI Data Acquisition

All subjects underwent neuropsychological tests, comprising the following assessments: to evaluate general cognition, the Mini-Mental State Examination and the Montreal Cognitive Assessment Scale were used; to evaluate memory function, the delayed recall test of Webster's Memory Scale–Logical Memory II and the immediate and delayed memory parts of the Auditory Verbal Learning Test (AVLT) were used; to evaluate executive function, parts A and B of the Trail-Making Test were used; to evaluate daily cognition, the Everyday Cognition test (patient and informant version) was used; and to evaluate visuospatial function, the Clock-Drawing Test was used.

Magnetization-prepared rapid gradient echo (MPRAGE), 3D T_1_-weighted image (T_1_WI), and echo-planar imaging (EPI) rs-fMRI sequences were included in this study. The parameters of each sequence were as follows: sagittal MPRAGE 3D-T_1_WI: repetition time (TR)/echo time (TE) = 2,300 ms/2.95 ms; field of view = 240 × 256 mm; flip angle = 9°; thickness = 1.2 mm; 176 slices; and EPI rs-fMRI: TR/TE = 3,000 ms/30 ms; spatial resolution = 3.4 × 3.4 × 3.4 mm; 48 slices; 197 time points. All subjects were instructed to keep their eyes open as normal and to rest calmly during the scan.

AVLT, Auditory Verbal Learning Test; CDT, Clock Drawing Test; ECog, Everyday Cognition scale; MCI, mild cognitive impairment; MMSE, Mini-Mental State Examination; MoCA, Montreal Cognitive Assessment Scale; NC, normal control; SCD, subjective cognitive decline; TMT-A and -B, Trail-Making Test, parts A and B; WMS-LM II, Webster's Memory Scale–Logical Memory II.

### MRI Data Preprocessing and Calculation of rs-fMRI Indices

**Figure 1:**
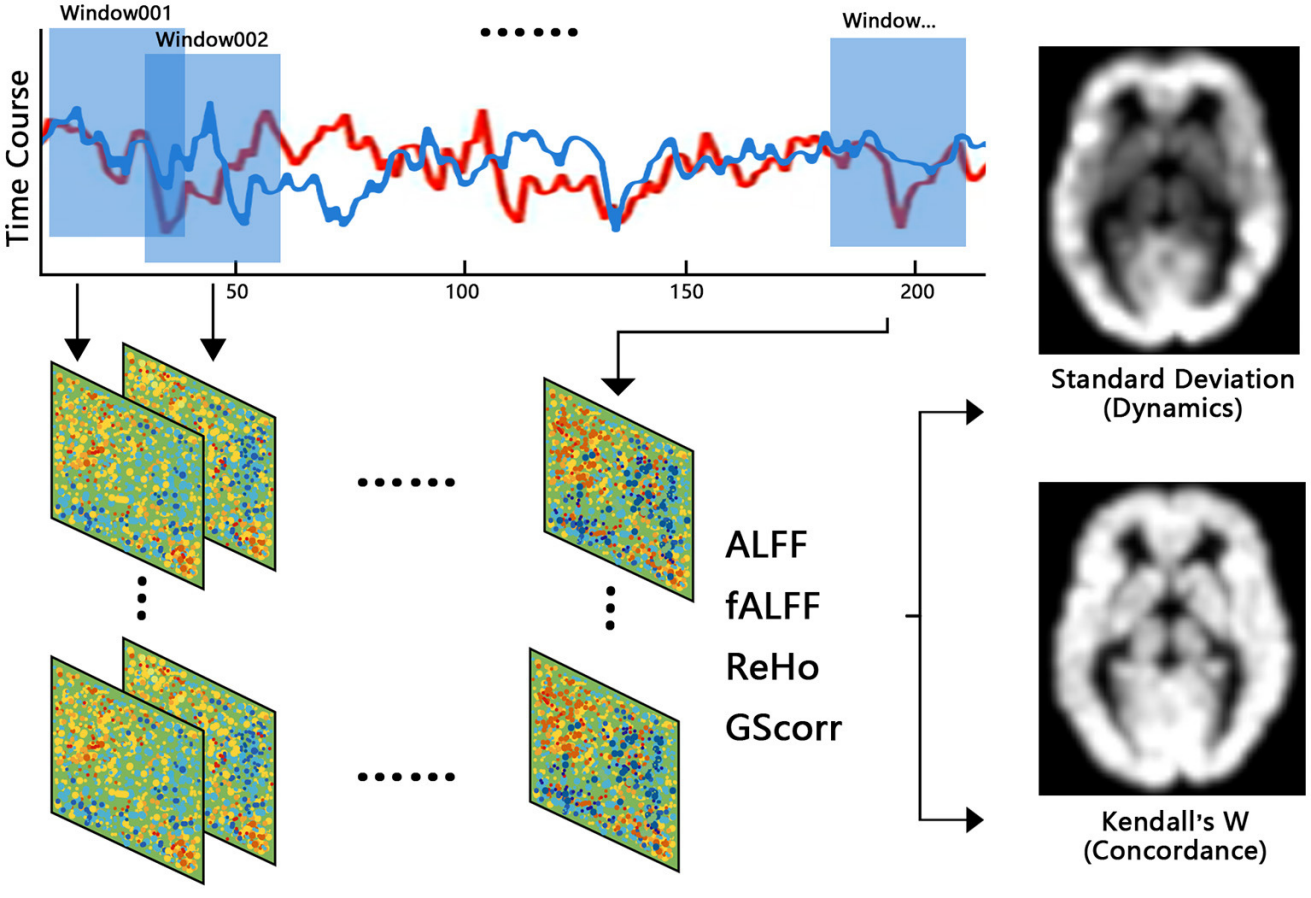
Brief information about dynamic and concordance indices calculation.

Image preprocessing was performed using the Data Processing Assistant for Resting-State fMRI toolbox (DPARSF; http://rfmri.org/DPARSF). The first 10 volumes were removed to allow for adaptation of participants to the environment, and the remaining 187 volumes were corrected for timing differences. The corrected functional sequences from each subject were motion-corrected using a six-parameter (rigid body) linear transformation. Forty subjects were removed based on exclusion criteria of head movements leading to >2-mm translation or >2° rotation in any direction. Next, individual T_1_WI images were coregistered to the mean functional images and then segmented into gray matter, WM, and cerebrospinal fluid (CSF). Based on these segmented images, the functional volumes of each individual were spatially normalized to the Montreal Neurological Institute space using the Diffeomorphic Anatomical Registration Through Exponentiated Lie Algebra (DARTEL) toolbox and then resampled to 3-mm isotropic voxels. To prepare the data for extracting rs-fMRI indices, functional volumes were selectively smoothed with a 6-mm full width at half maximum (FWHM) Gaussian kernel, and the linear trend of the time course was removed, or the volumes were filtered using a bandpass filter of 0.01–0.08 Hz. In order to eliminate the influence of FWHM on the results, we also adopted FWHM kernel of 4 and 8 mm. Finally, nuisance signals including Friston 24-head motion parameters and CSF signals were extracted and regressed out from the data to reduce the effects of non-neuronal signals. For studies that have dispute over whether the mean global and WM signal regress (Murphy and Fox, 2017; Ding et al., 2018; Li et al., 2019a), we have studied the data with removal and non-removal of the mean global signal and WM signal, respectively. The resultant residual time series were used for further analyses (Figure 1).

### Calculation of rs-fMRI Indices of Dynamics

Sliding window analysis was used to generate rs-fMRI indices of brain dynamics using the Temporal Dynamic Analysis toolkit in the Data Processing & Analysis of Brain Imaging (DPABI; http://rfmri.org/DPABI) toolbox. Moderate Hamming windows with a length of 30 TR and an overlap of 1 TR were applied to the preprocessed time series to obtain windowed time series. For each subject, 187 postprocessed volumes were segmented into 158 windows in total. Within each window, the following indices of IBA were calculated: (1) ALFF, (2) fractional ALFF (fALFF), (3) regional homogeneity (ReHo), and (4) global signal correlation (GSCorr). It is worth noting that data for ALFF and fALFF were smoothed but not filtered, and that data for the other indices were filtered but not smoothed. Standard deviation (SD) maps of each index across the windows were later calculated to characterize the dynamics of the rs-fMRI indices (yielding dALFF, dfALFF, dReHo, and dGSCorr). Finally, *Z* standardization and smoothing (except for dALFF and dfALFF, which were smoothed during preprocessing) were performed on these SD maps. Other window sizes of 60 and 90 TR were also conducted in this study.

### Concordance Index of rs-fMRI Indices

The concordance indices were calculated based on Kendall's *W* among each rs-fMRI index. We calculated two kinds of concordance index: (1) the volume-wise concordance index, with Kendall's *W* of the rs-fMRI indices across all brain voxels for each subject across all time windows during the scan, was calculated as the global-level concordance index; (2) the voxel-wise concordance index, with Kendall's *W* for the rs-fMRI indices of each voxel for each subject across time windows was calculated as the voxel-level concordance index. Considering the high similarity between ALFF and fALFF, and fALFF is less susceptible to artifactual contributions of motion and pulsatile effects compared to ALFF (Yan et al., 2017), only the fALFF, ReHo, and GSCorr indices were used to calculate the concordance index in this study, in order to avoid artificially improving the concordance.

### Statistical Analysis

Data regarding the gender and genetics of subjects were compared among groups using the χ^2^-test. The demographic data, neuropsychological scale scores, and volume-wise concordance indices of each group were analyzed using one-way analyses of variance (ANOVAs), with the least significant difference (LSD) test used for *post-hoc* comparisons. *P* < 0.05 was considered as statistically significant. One-way ANOVAs were performed to compare the standardized SD maps of each rs-fMRI index and the voxel-wise concordance maps among the three groups. In order to reduce the influence of factors such as age, gender, education, head motion, and scanning site on the results, these factors were used as covariates and removed by regression during statistical analysis. Gaussian random field correction was used to correct for multiple comparisons, using a voxel-level *P* < 0.001 and cluster-level *P* < 0.05. Finally, the concordance indices in regions with significant differences in each SCD individual were extracted, and correlations between the concordance indices of each brain area and scores on the neuropsychological scales were analyzed using a general linear model:

$$\begin{array}{l} Y=\beta_{0}+\beta_{1}\times V_{\text{scores}}+\beta_{2}\times V_{\text{age}}+\beta_{3}\times V_{\text{sex}}+\beta_{4}\times V_{\text{edu}}\\ \;+\beta_{5}\times V_{\text{meanFD}}+\beta_{6}\times V_{\text{site}}+\text{error} \end{array}$$

## Results

### Demographic and Neuropsychological Data

There were no significant differences in age, gender, and education level among the SCD, MCI, and NC groups (*P* > 0.05) (Table 1), whereas difference in *APOE* ε*4* status of the three groups was significant (*P* = 0.046). In contrast, all neuropsychological test scores differed significantly among the three groups (*P* < 0.05, Table 1). LSD *post-hoc* analysis revealed that only the AVLT immediate memory scores (*P* = 0.033) were significantly lower in the SCD group than those in NCs; all of the other significant neuropsychological differences resulted from comparisons between the MCI group and the other two groups, respectively (*P* < 0.001) (Supplementary Table 1).

**Table 1:** Comparison of demographic and neuropsychological data among the SCD, MCI, and NC groups.

	**NC (*n* = 82)**	**SCD (*n* = 94)**	**MCI (*n* = 75)**	***F*/χ^**2**^**	***P***
**Demographics**					
Gender (female/male)	49/33	60/34	35/40	5.308	0.070
*APOE ε4* (+/–)	19/61	33/47	26/42	6.140	0.046
Age (years)	73.88 ± 7.40	71.07 ± 6.18	74.36 ± 8.42	1.680	0.191
Education (years)	16.70 ± 2.18	16.91 ± 2.17	16.03 ± 2.84	0.792	0.454
Jenkinson mean FD	0.11 ± 0.07	0.12 ± 0.06	0.12 ±0.07	0.770	0.680
**Global cognition**					
MMSE	28.88 ± 1.53	29.03 ± 1.14	26.96 ± 3.71	19.574	<0.001
MoCA	26.40 ± 2.78	26.03 ± 2.65	22.37 ± 4.10	37.441	<0.001
**Memory**					
WMS-LM II delayed recall	14.13 ± 3.67	13.05 ± 3.89	7.20 ± 4.48	66.965	<0.001
AVLT immediate recall	9.23 ± 4.78	7.76 ± 4.40	4.35 ± 4.20	23.982	<0.001
AVLT delayed recall	13.48 ± 1.97	12.92 ± 2.40	10.72 ± 3.63	22.274	<0.001
**Executive function**					
TMT-A (s)	31.07 ± 9.68	30.71 ± 8.80	41.78 ± 27.18	11.091	<0.001
TMT-B (s)	74.98 ± 40.53	74.64 ± 28.12	109.19 ± 75.69	12.039	<0.001
**Everyday cognition**					
ECog memory (patient)	1.40 ± 0.48	1.82 ± 0.54	2.30 ± 0.80	41.363	<0.001
ECog total (patient)	1.23 ± 0.30	1.52 ± 0.41	1.84 ± 0.61	34.824	<0.001
ECog memory (informant)	1.28 ± 0.39	1.40 ± 0.51	2.28 ± 0.91	56.833	<0.001
ECog total (informer)	1.14 ± 0.22	1.22 ± 0.30	1.88 ± 0.76	55.901	<0.001
**Visuospatial function**					
CDT	4.66 ± 0.67	4.77 ± 0.51	4.33 ± 1.10	6.966	0.001

### Dynamics of rs-fMRI indices

**Figure 2:**
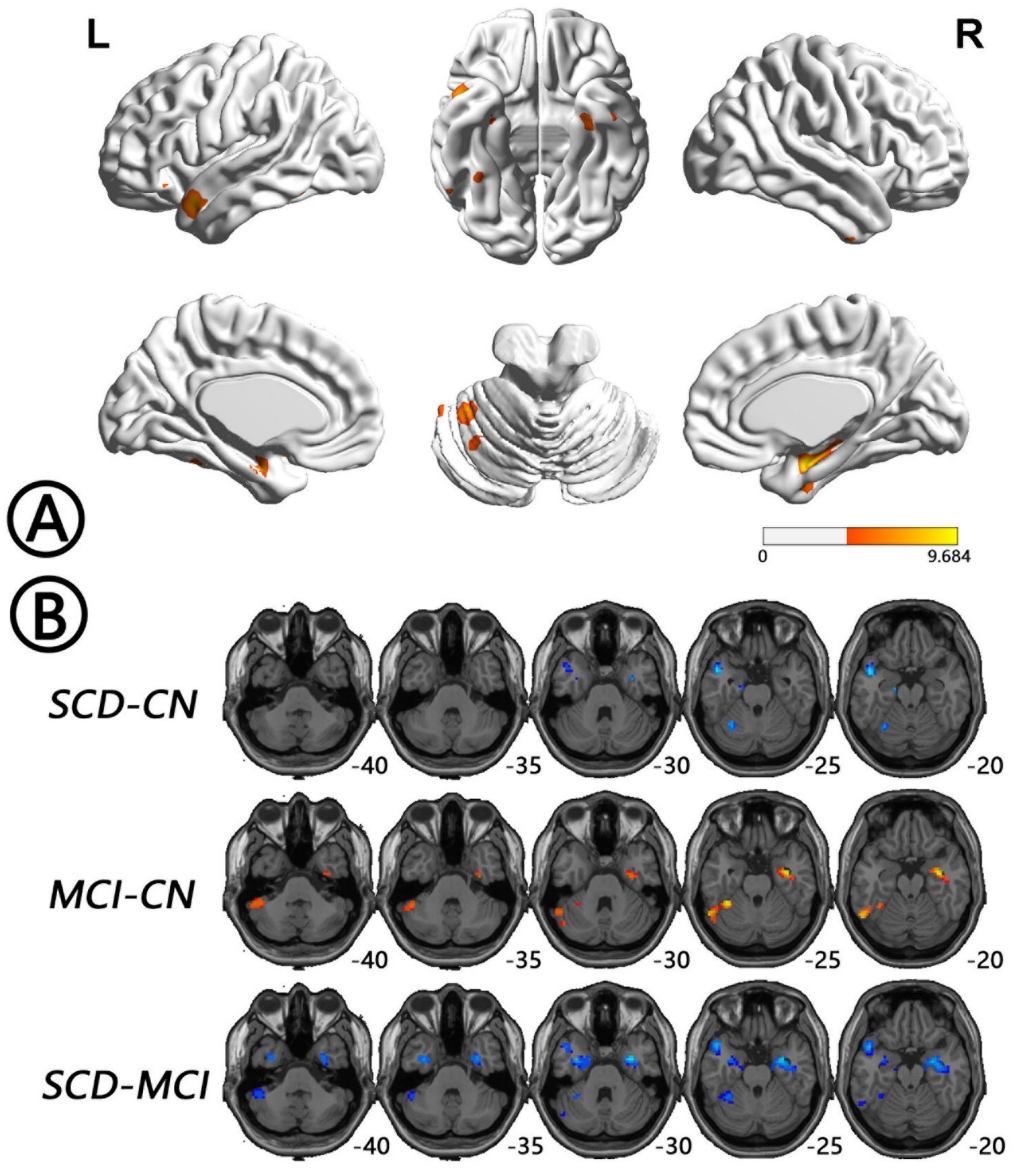
Regions with differences in dALFF between the SCD, MCI, and NC groups and *post-hoc* analysis brain maps (GRF-corrected, voxel-level *P* < 0.001, cluster-level *P* < 0.05). **(A)** Differences in dALFF were shown in bilateral HP/PHG/FG, which extended to the left STG/MTG/TP and bilateral cerebellum among groups (red). **(B)** dALFF values in the MCI group were higher than those in the NC group (red), whereas those in the SCD group were lower than those in the other two groups (blue). dALFF, dynamics of amplitude of low-frequency fluctuations; FG, fusiform gyrus; GRF, Gaussian random field; HP, hippocampus; MCI, mild cognitive impairment; MTG, middle temporal gyrus; NC, normal control; PHG, parahippocampal gyrus; SCD, subjective cognitive decline; STG, superior temporal gyrus; TP, temporal pole.

**Figure 3:**
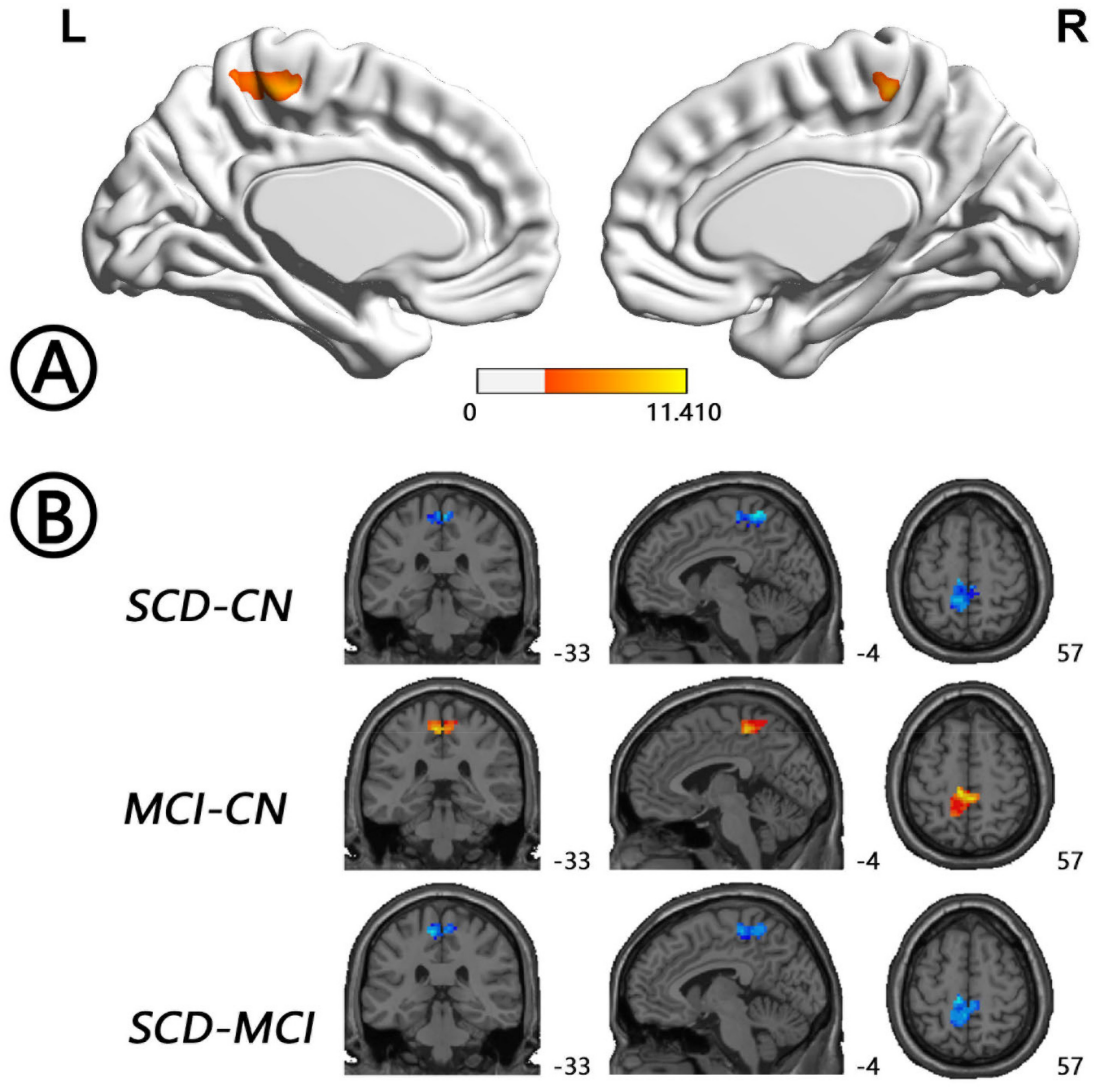
Regions with differences in dfALFF between the SCD, MCI, and NC groups and *post-hoc* analysis brain maps (GRF-corrected, voxel-level *P* < 0.001, cluster-level *P* < 0.05). **(A)** Differences in dfALFF were shown in bilateral PreCu and paracentral lobule (red). **(B)** dfALFF values in the MCI group were higher than those in the NC group (red), whereas those in the SCD group were lower than those in the other two groups (blue). dfALFF, dynamics of fractional amplitude of low-frequency fluctuations; GRF, Gaussian random field; MCI, mild cognitive impairment; NC, normal control; PreCu, precuneus; SCD, subjective cognitive decline.

**Figure 4:**
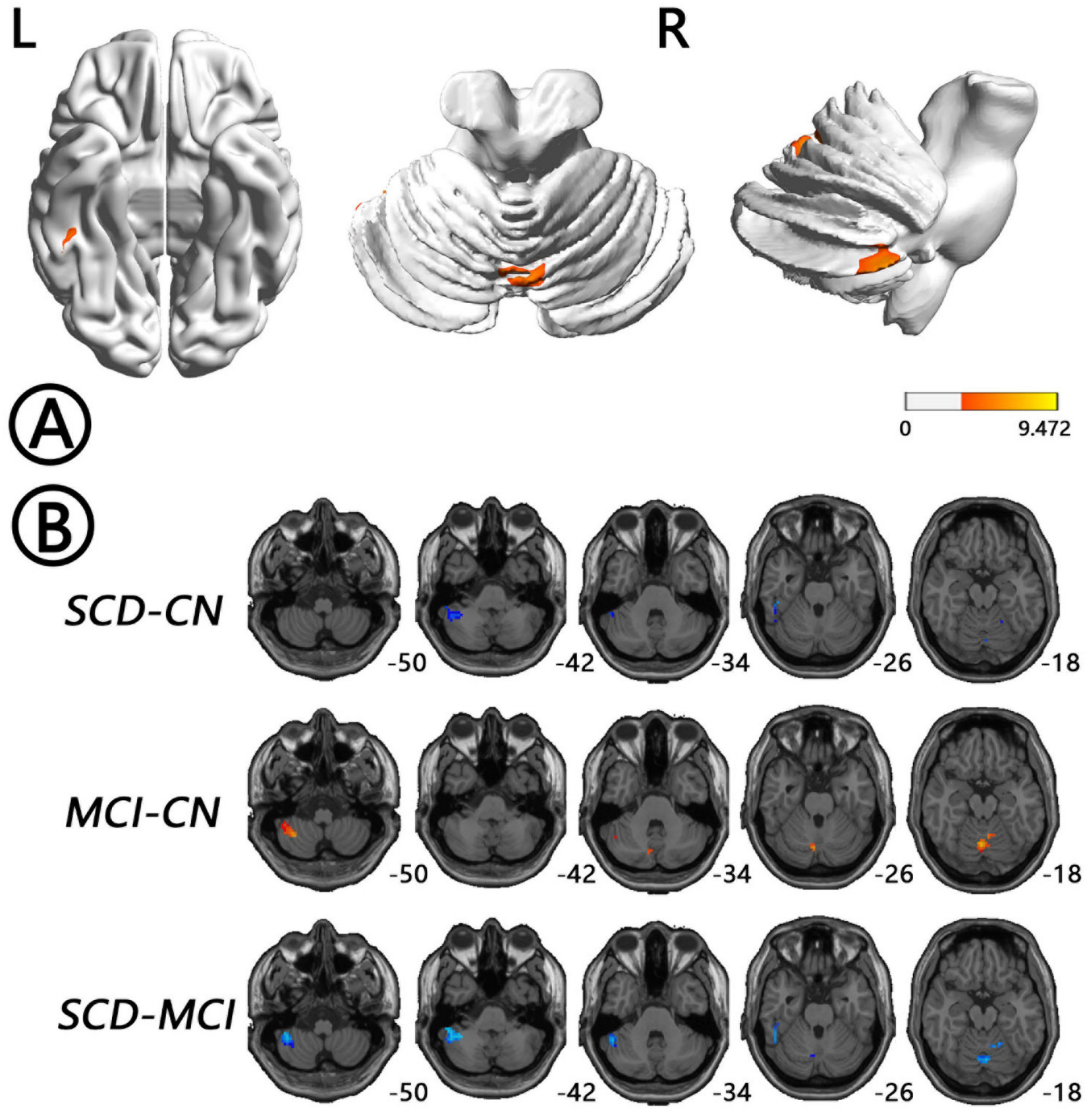
Regions with differences in dReHo between the SCD, MCI, and NC groups and *post-hoc* analysis brain maps (GRF-corrected, voxel-level *P* < 0.001, cluster-level *P* < 0.05). **(A)** Differences in dReHo were shown in left cerebellum posterior lobe, which extended to the left FG, vermis, and right cerebellum anterior lobe (red). **(B)** dReHo values in the MCI group were higher than those in the NC group (red), whereas those in the SCD group were lower than those in the other two groups (blue). dReHo, dynamics of regional homogeneity; FG, fusiform gyrus; GRF, Gaussian random field; MCI, mild cognitive impairment; NC, normal control; SCD, subjective cognitive decline.

The intergroup differences in ALFF dynamics (dALFF) included bilateral HP/parahippocampal gyrus (PHG)/FG, which extended to the left superior temporal gyrus/middle temporal gyrus)/temporal pole (TP), and bilateral cerebellum. Differences in fALFF dynamics (dfALFF) among the three groups included bilateral PreCu and paracentral lobule. And differences in ReHo dynamics (dReHo) among the three groups included left cerebellum posterior lobe, which extended to the left FG, vermis, and right cerebellum anterior lobe. There were no significant differences in dGSCorr among the three groups. *Post-hoc* analysis using LSD testing showed that dALFF, dfALFF, and dReHo in the MCI group were higher than those in the NC group, whereas these measures were lower in the SCD group than those in the other two groups (Figures 2–4, Table 2). All of the results were preserved when we omitted different FWHM Gaussian kernel or different Hamming window size (Supplementary Figures 1–4). Results of whether the WM or mean global signal regresses were similar (Supplementary Figures 5, 6).

**Table 2:** Regions with differences in rs-fMRI indices of dynamics among the SCD, MCI, and NC groups.

**Brain regions**	**Brodmann area**	**Size (voxels)**	**MNI coordinates (mm)**			**Peak value**
			***X***	***Y***	***Z***	
**dALFF**						
Right HP/PHG/FG	20/28/36	281	30	−6	−24	7.0327
Left HP/PHG/FG extend to STG/MTG/TP	20/21/36/38	270	−51	12	−21	9.6840
Left cerebellum anterior/ posterior lobe	—	226	−37	−48	−23	7.2869
**dfALFF**						
Bilateral PreCu/paracentral lobule	5	184	1	−37	54	11.2712
**dReHo**						
Left cerebellum posterior lobe extend to FG	—	190	−42	−48	−48	6.2432
Right cerebellum anterior lobe and vermis	—	104	−3	−63	−15	8.1379

*dALFF, dynamics of amplitude of low-frequency fluctuations; dfALFF, dynamics of fractional amplitude of low-frequency fluctuations; dReHo, dynamics of regional homogeneity; FG, fusiform gyrus; HP, hippocampus; MNI, Montreal Neurological Institute; MTG, middle temporal gyrus; PHG, parahippocampal gyrus; PreCu, precuneus; STG, superior temporal gyrus; TP, temporal pole*.

### Concordance of rs-fMRI Indices

**Figure 5:**
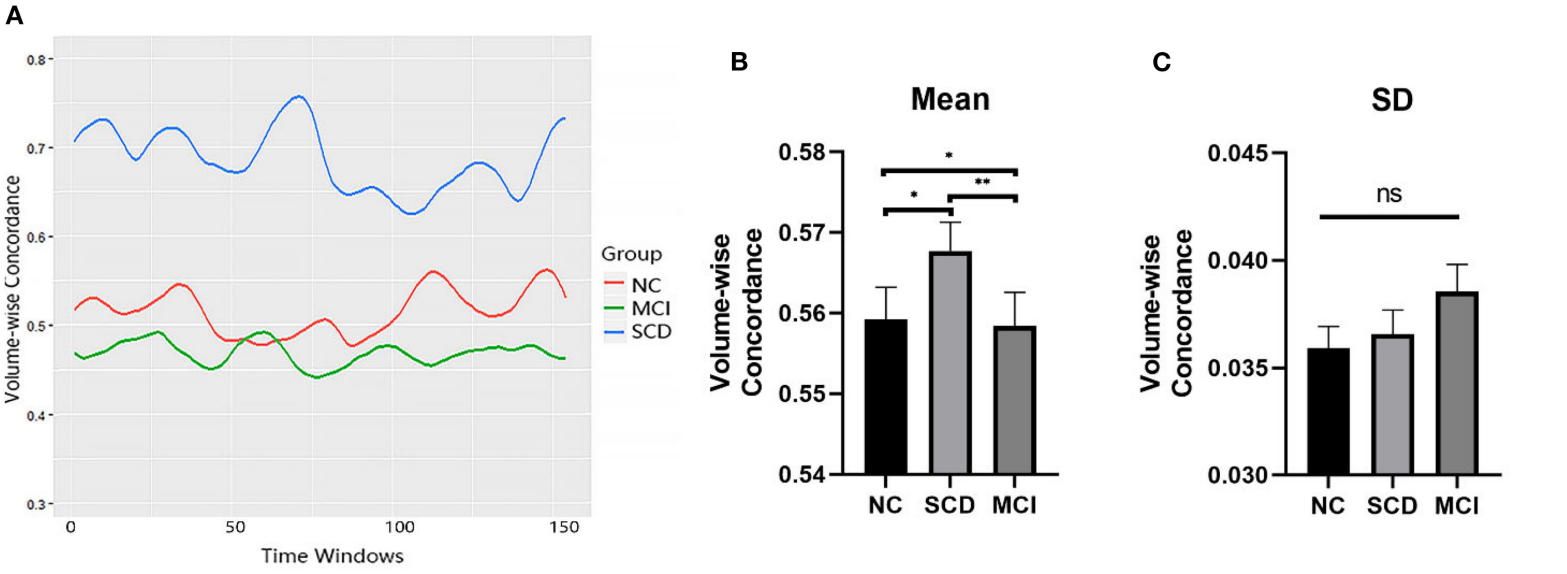
Comparison of volume-wise concordance indices among the SCD, MCI, and NC groups. **(A)** Volume-wise concordance time series of typical SCD, MCI, and NC subjects. **(B)** Comparison of mean volume-wise concordance indices. **(C)** Comparison of the SD of volume-wise concordance indices. MCI, mild cognitive impairment; NC, normal control; SCD, subjective cognitive decline; SD, Standard deviation. *P < 0.05; **P < 0.01; ns, no significance.

The mean values of the volume-wise concordance in the SCD, MCI, and NC groups show significant differences (*P* = 0.005). Subsequent analyses showed that the mean concordance of the MCI group was lower than that of the NC group (*P* = 0.035), whereas that of the SCD group was higher than those of the MCI (*P* = 0.002) and NC groups (*P* = 0.022). There was no statistical difference in the SD of the volume-wise concordance among the three groups (*P* = 0.445) (Figure 5, Table 3).

**Table 3:** Comparison of volume-wise concordance indices among the SCD, MCI, and NC groups.

	**NC**	**SCD**	**MCI**	***F***	***P***
Mean	0.56 ± 0.04	0.57 ± 0.03	0.55 ± 0.03	5.510	0.005
SD	0.04 ± 0.01	0.04 ± 0.01	0.04 ± 0.01	0.811	0.445

*MCI, mild cognitive impairment; NC, normal control; SCD, subjective cognitive decline; SD, standard deviation*.

**Figure 6:**
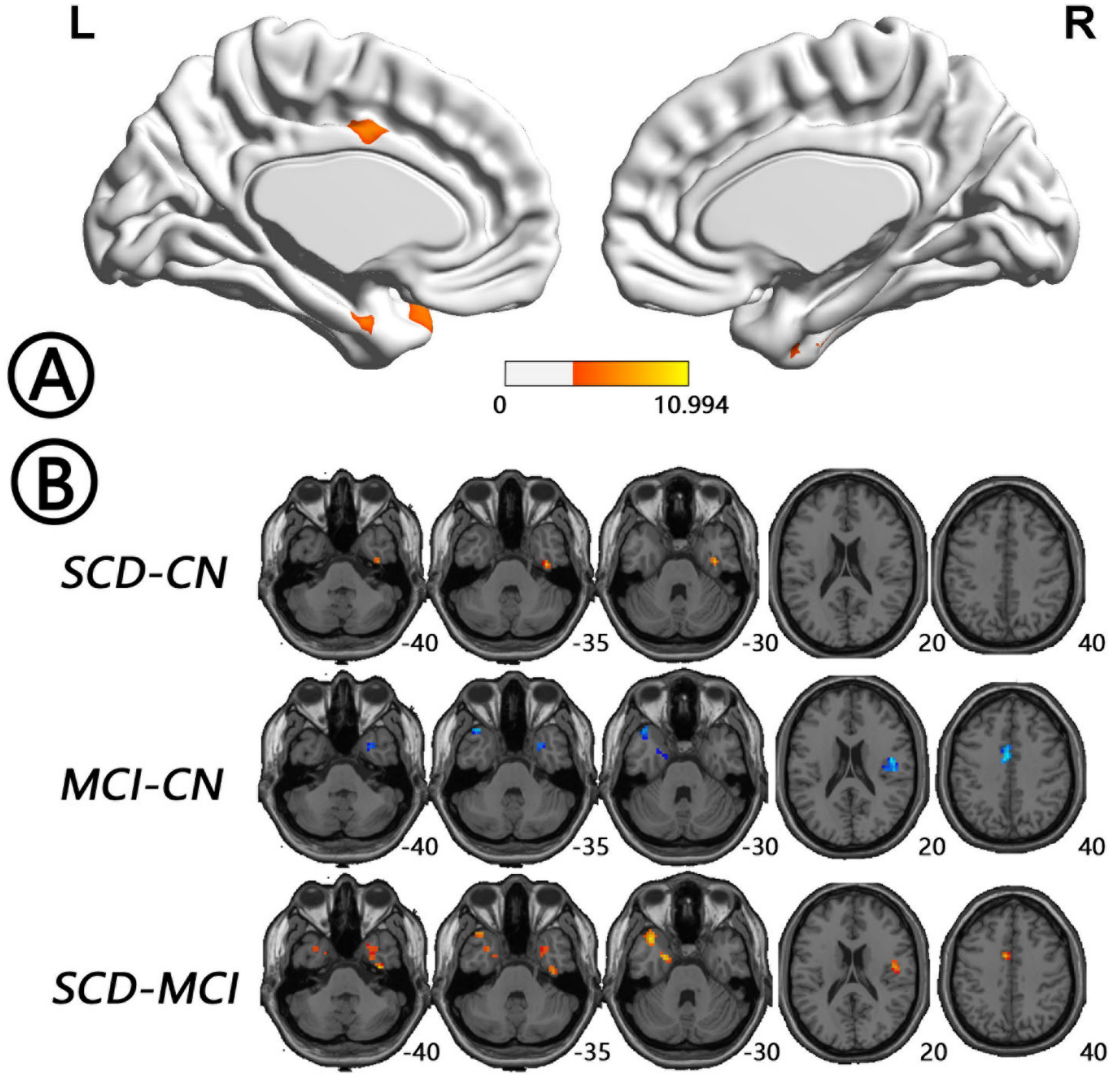
Regions with differences in voxel-wise concordance between the SCD, MCI, and NC groups and *post-hoc* analysis brain maps (GRF-corrected, voxel-level *P* < 0.001, cluster-level *P* < 0.05). **(A)** Concordance differences were found in left HP/PHG, left insula/TP, left MCC, right TP/FG, right Rolandic operculum (RO)/insula (red). **(B)** The concordance of these regions in the MCI patients was lower than that in the NCs (blue), whereas the concordance in SCD patients was higher than that in the other two groups (red). FG, fusiform gyrus; GRF, Gaussian random field; HP, hippocampus; MCC, midcingulate cortex; MCI, mild cognitive impairment; NC, normal control; PHG, parahippocampal gyrus; RO, Rolandic operculum; SCD, subjective cognitive decline; TP, temporal pole.

Comparing the voxel-wise concordance maps among the three groups, significant differences were found in left HP/PHG, left insula/TP, left midcingulate cortex (MCC), right TP/FG, and right Rolandic operculum/insula. Subsequent analysis showed that the concordance of these regions in the MCI patients was lower than that in the NCs, whereas the concordance in SCD patients was higher than those in the other two groups (Figure 6, Table 4). All of the results were preserved with different FWHM Gaussian kernel or different Hamming window size (Supplementary Figures 7, 8). Results of whether the WM or mean global signal regresses were similar as well (Supplementary Figure 9).

**Table 4:** Regions with differences in the concordance of rs-fMRI indices among the SCD, MCI, and NC groups.

**Brain regions**	**Brodmann area**	**Size (voxels)**	**MNI coordinates (mm)**			**Peak value**
			***X***	***Y***	***Z***	
Left PHG/HP	28/36	53	−18	−3	−27	7.4466
Left MCC	23/24	62	−6	−6	39	6.6607
Left insula/TP	38/48	133	−44	10	−12	7.3861
Right FG/TP	20/36	89	40	−15	−32	6.7529
Right RO/insula	48	58	45	−9	21	8.9552

*FG, fusiform gyrus; HP, hippocampus; MCC, midcingulate cortex; MNI, Montreal Neurological Institute; PHG, parahippocampal gyrus; RO, Rolandic operculum; TP, temporal pole*.

No correlation between concordance values and neuropsychological scale scores was found in the SCD.

## Discussion

In this study, we found that SCD individuals had lower dALFF and dReHo values and higher concordance in memory-related brain regions such as HP, PHG, and FG compared with the other two groups. MCI patients showed high dynamics-related indices in these areas, suggesting insufficient stability of IBA in these areas. In contrast, SCD individuals showed IBA dynamics values that were even lower than those in the NC group, indicating stable but inflexible brain activity. This alteration might affect the efficient, rapid, and complex processes related to memory coding, resulting in memory loss in SCD patients. The concordance of rs-fMRI indices reflects the integrative function of the brain (Yan et al., 2017). Concordance in MCI patients was significantly lower than that in NCs, indicating that this integrative function might be impaired in MCI patients or in those who suffer from a cognitive disorder. However, the concordance was high in SCD individuals, suggesting that their integrative function was intact; indeed, it was even higher than that in NCs, which is not consistent with the memory loss observed in SCD individuals. We infer that this finding might reflect ineffective integration or be the result of a compensatory mechanism (Elman et al., 2014).

Brain regions showing differences in dALFF among the three groups were more extensive, including bilateral HP/PHG/FG and bilateral cerebellum. All these regions are consistent with brain areas reported for tau protein deposition in AD patients (Congdon and Sigurdsson, 2018). HP and PHG are essential structures of the medial temporal lobe and play a role in the coding of episodic memory (Eichenbaum et al., 2007). Visible HP and PHG atrophy can be found in MCI and AD patients (Yang et al., 2012), and furthermore, the functional connectivity between HP, PHG, and other brain regions has been shown to be reduced in MCI and AD patients (Liu et al., 2015). The FG is the center of face recognition (Weiner and Zilles, 2016), with recent studies showing that FG also plays a vital role in semantic memory (Forseth et al., 2018) and that abnormalities in left FG can result in semantic dementia (Merck et al., 2017). In this study, we found increased dynamics in bilateral HP/PHG/FG in SCD patients, which is consistent with results of Li et al. (2018). In addition to dALFF, dReHo also showed differences among groups in the bilateral cerebellum. The cerebellum is the center of motor regulation and forms a cognitive and motor loop with the telencephalon (Salmi et al., 2010), with both cerebellum (Jacobs et al., 2018; Schmahmann, 2019) and vermis (Wang et al., 2017) critical to cognitive function. Abnormal IBA in cerebellar regions has been reported in SCD, MCI, and AD patients (Yang et al., 2012; Sun et al., 2016). Besides, bilateral PreCu also showed dfALFF differences among groups. PreCu locates in the posteromedial portion of the parietal lobe. Functional imaging findings in healthy subjects suggest a central role for the PreCu in a wide spectrum of highly integrated tasks, including episodic memory retrieval (Cavanna and Trimble, 2006). The PreCu and PCC regions together constitute a key hub of the default mode network, which is also an area associated with the accumulation of amyloid-β plaque (Buckner et al., 2009). The PCC/PreCu exhibits reduced functional connectivity in mild AD patients (Zhang et al., 2009). After 12 weeks of moderate-intensity walking exercise training, functional connectivity of the PCC/PreCu was increased in individuals with MCI (Chirles et al., 2017).

Our results showed intergroup concordance differences in left HP/PHG, MCC, and bilateral temporal lobe regions. The temporal lobe is involved in memory and language function (Eichenbaum et al., 2007), whereas the cingulate cortex plays a key role in cognitive, motor, and emotional function, with activity in MCC correlating with cognitive and sensorimotor networks (Kragel et al., 2018). Studies have shown significant atrophy of the temporal lobe in patients with MCI (Sheelakumari et al., 2018; DeVivo et al., 2019). Moreover, the global functional connectivity of the cingulate cortex, including MCC, shows deterioration in MCI patients (Cera et al., 2019).

No correlation between concordance values and neuropsychological scale scores was found in the SCD group in our research. This may be that the cognitive changes of SCD individuals caused by abnormal functional integration ability were too subtle, and simple cognitive scales cannot reflect such subtle changes. Therefore, we need to find a scale that can identify SCD individuals more efficiently.

There are several limitations to this study. First, the sample size was insufficient; thus, further analyses with more participants are warranted. Second, ADNI is a multicenter study, and although the influence of site was regressed out as a covariate in our statistical analyses, its effect still cannot be removed completely. Third, only ALFF, fALFF, ReHo, and GSCorr indices were used in this study, and more parameters could be analyzed in future studies. Finally, the sliding window method in this study requires a fixed window width, but in the future, a more comprehensive analysis without a fixed window width could be conducted.

SCD individuals showed both alterations to the dynamics and concordance of their IBA. These results suggest that analysis of temporal dynamics based on rs-fMRI data is a novel approach for investigating brain alterations in individuals with SCD. Furthermore, these alterations in dynamics and concordance might help us to better understand the mechanisms underlying brain activity changes in SCD individuals.

## Data Availability Statement

The datasets presented in this study can be found in online repositories. The names of the repository/repositories and accession number(s) can be found in the article/Supplementary Material.

## Ethics Statement

The studies involving human participants were reviewed and approved by Alzheimer's Disease Neuroimaging Initiative Coordinating Center. The patients/participants provided their written informed consent to participate in this study.

## Author Contributions

YY contributed to the design the work, analyzed the data, and wrote the manuscript. XZhan provided technical support for the data analysis and revised the manuscript. XZha agreed to be accountable for all aspects of the work. JK provided technical support for the data analysis and revised the manuscript. SH gave final approval of the version to be published. XW agreed to be accountable for all aspects of the work. YS revised the manuscript and gave final approval of the version to be published. CH agreed to be accountable for all aspects of the work. All authors contributed to the article and approved the submitted version.

## Conflict of Interest

The authors declare that the research was conducted in the absence of any commercial or financial relationships that could be construed as a potential conflict of interest.
