# Supplementary material for: Dynamics and Concordance Abnormalities Among Indices of Intrinsic Brain Activity in Individuals With Subjective Cognitive Decline: A Temporal Dynamics Resting-State Functional Magnetic Resonance Imaging Analysis
Source: Front Aging Neurosci. 2021 Jan 25;12:584863. doi: 10.3389/fnagi.2020.584863 (PMC7868384; doi:10.3389/fnagi.2020.584863)
Supplement: Supplementary file 1 [file Table_1.DOCX]

Supplementary Material

# Inclusion criteria for individuals

The inclusion criteria for SCD (SMC in ADNI) individuals included: (a) having a self-reported memory decline; (b) Cognitive Change Index (CCI)≥ 16; (c) Wechsler Memory Scale Logical Memory (WMS-LM) Ⅱ delayed recall score is normal (a. ≥ 9 for 16 or more years of education; b. ≥ 5 for 8-15 years of education; c. ≥ 3 for 0-7 years of education); (d) Mini-Mental State Examination (MMSE) score ≥ 24；(e) clinical dementia rating (CDR) score = 0. Inclusion criteria for MCI patients included: (a) having a memory decline; (b) WMS-LM Ⅱ delayed recall score is lower than the normal range; (c) MMSE score ≥ 24；(d) clinical dementia rating (CDR) score = 0.5; (e) cognitive function does not meet the diagnostic criteria for AD. Inclusion criteria for NCs included: (a) having a normal memory performance; (b) the same as SCD (c)~(e).

# Supplementary Table 1

**The comparison of neuropsychological tests among SCD, MCI and NCs**

|  | SCD-NC | SCD-MCI | MCI-NC |
| --- | --- | --- | --- |
| MMSE | 0.658 | < 0.001 | < 0.001 |
| MOCA | 0.456 | < 0.001 | < 0.001 |
| WMS-LM Ⅱ delay recall | 0.076 | < 0.001 | < 0.001 |
| AVLT immediate recall | 0.033 | < 0.001 | < 0.001 |
| AVLT delayed recall | 0.184 | < 0.001 | < 0.001 |
| TMT-A（s） | 0.887 | < 0.001 | < 0.001 |
| TMT-B（s） | 0.965 | < 0.001 | < 0.001 |
| Ecog memory (patient) | < 0.001 | < 0.001 | < 0.001 |
| Ecog total (patient) | < 0.001 | < 0.001 | < 0.001 |
| Ecog memory (informer) | 0.211 | < 0.001 | < 0.001 |
| Ecog total (informer) | 0.289 | < 0.001 | < 0.001 |
| CDT | 0.348 | 0.009 | < 0.001 |

AVLT: Auditory Word Learning Test; CDT, Clock Drawing Test; Ecog: Everyday cognition scale; MCI: mild cognitive impairment; MMSE: Mini-Mental State Scale; MOCA: Montreal Cognitive Assessment Scale; NC: normal control; SCD: subjective cognitive decline; TMT-A, B: Trails-Making Test A, B; WMS-LM II: Webster’s Memory Scale-Logical Memory II.

# Supplementary figure 1


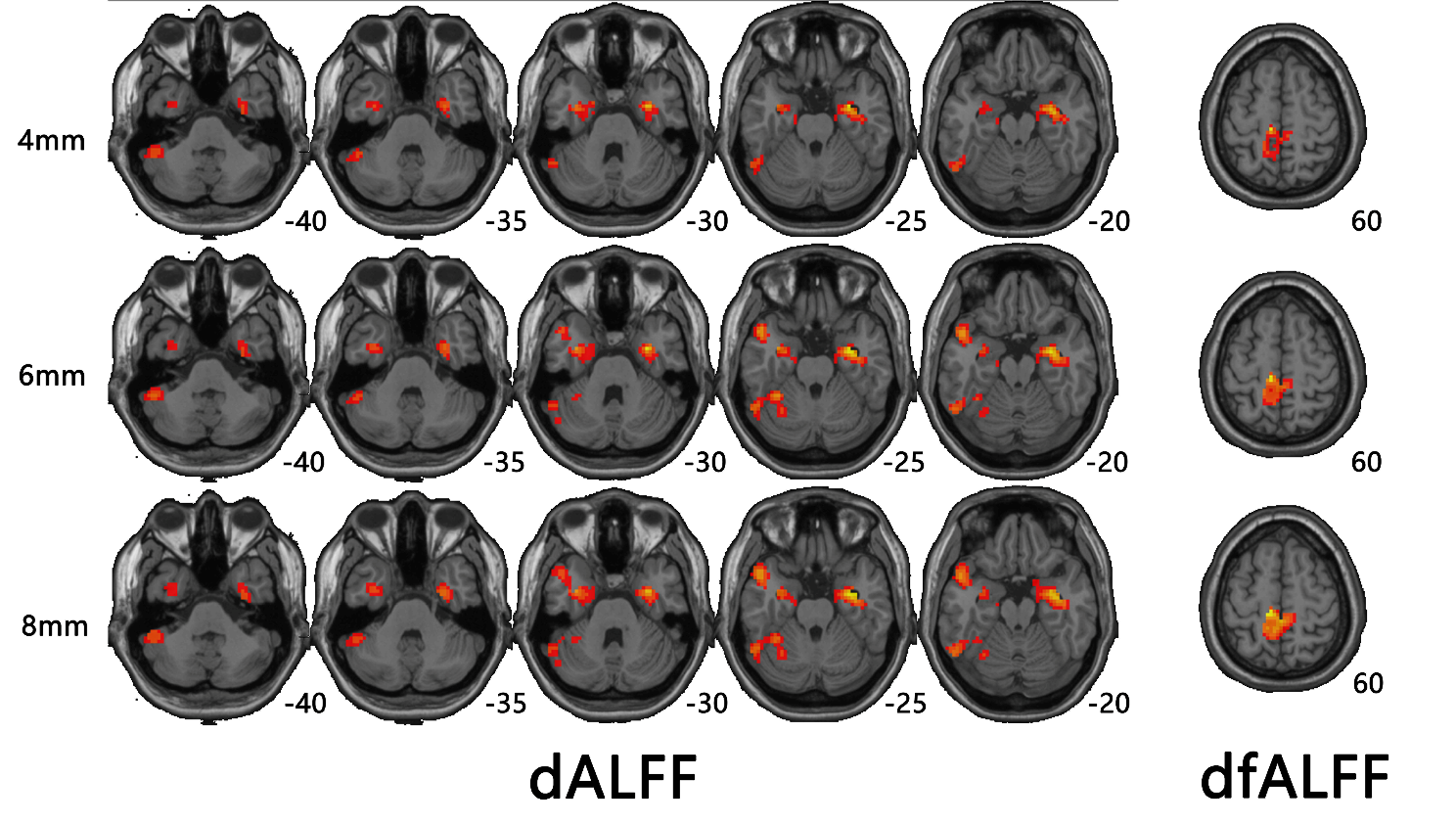


**Supplementary figure 1.** Differences in dALFF and dfALFF between the SCD, MCI, and NC groups with different FWHM Gaussian kernel. dALFF, dynamics of amplitude of low-frequency fluctuations; dfALFF, dynamics of fractional amplitude of low-frequency fluctuations; FWHM, full width at half maximum; MCI, mild cognitive impairment; NC, normal control; SCD, subjective cognitive decline.

# Supplementary figure 2


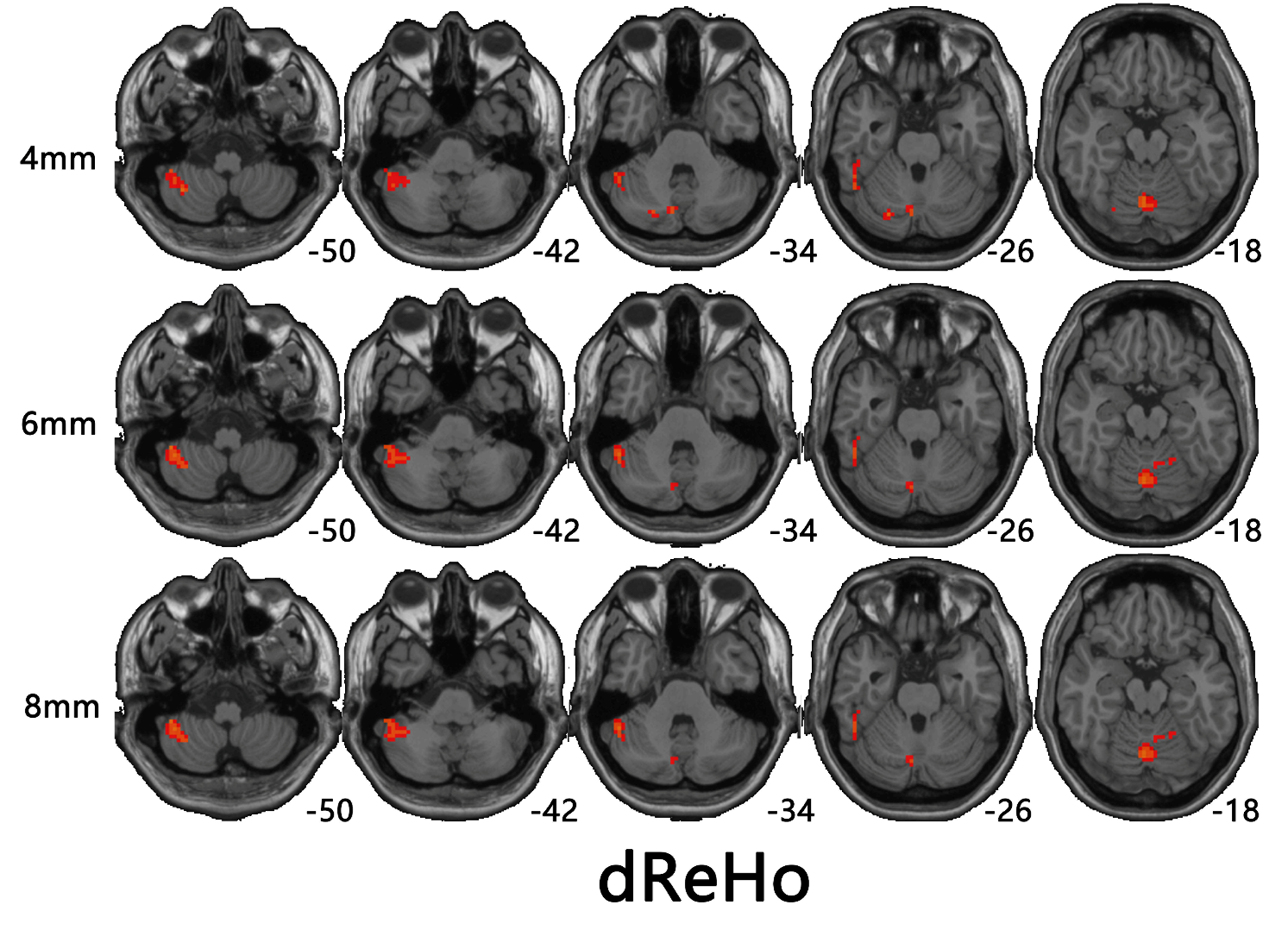


**Supplementary figure 2.** Differences in dReHo between the SCD, MCI, and NC groups with different FWHM Gaussian kernel. dReHo, dynamics of regional homogeneity; FWHM, full width at half maximum; MCI, mild cognitive impairment; NC, normal control; SCD, subjective cognitive decline.

# Supplementary figure 3


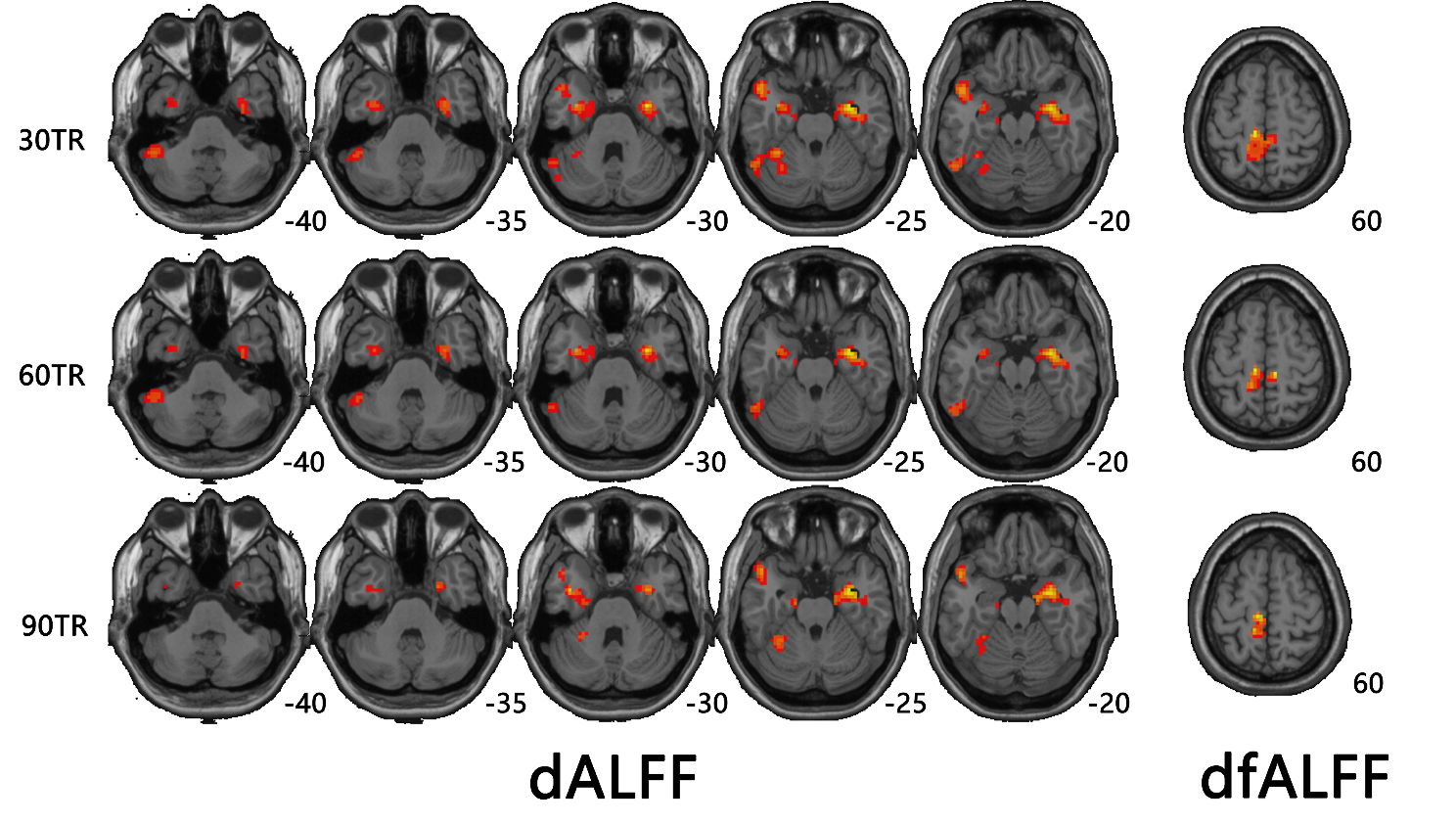


**Supplementary figure 3.** Differences in dALFF and dfALFF between the SCD, MCI, and NC groups with different Hamming window size. dALFF, dynamics of amplitude of low-frequency fluctuations; dfALFF, dynamics of fractional amplitude of low-frequency fluctuations; MCI, mild cognitive impairment; NC, normal control; SCD, subjective cognitive decline.

# Supplementary figure 4


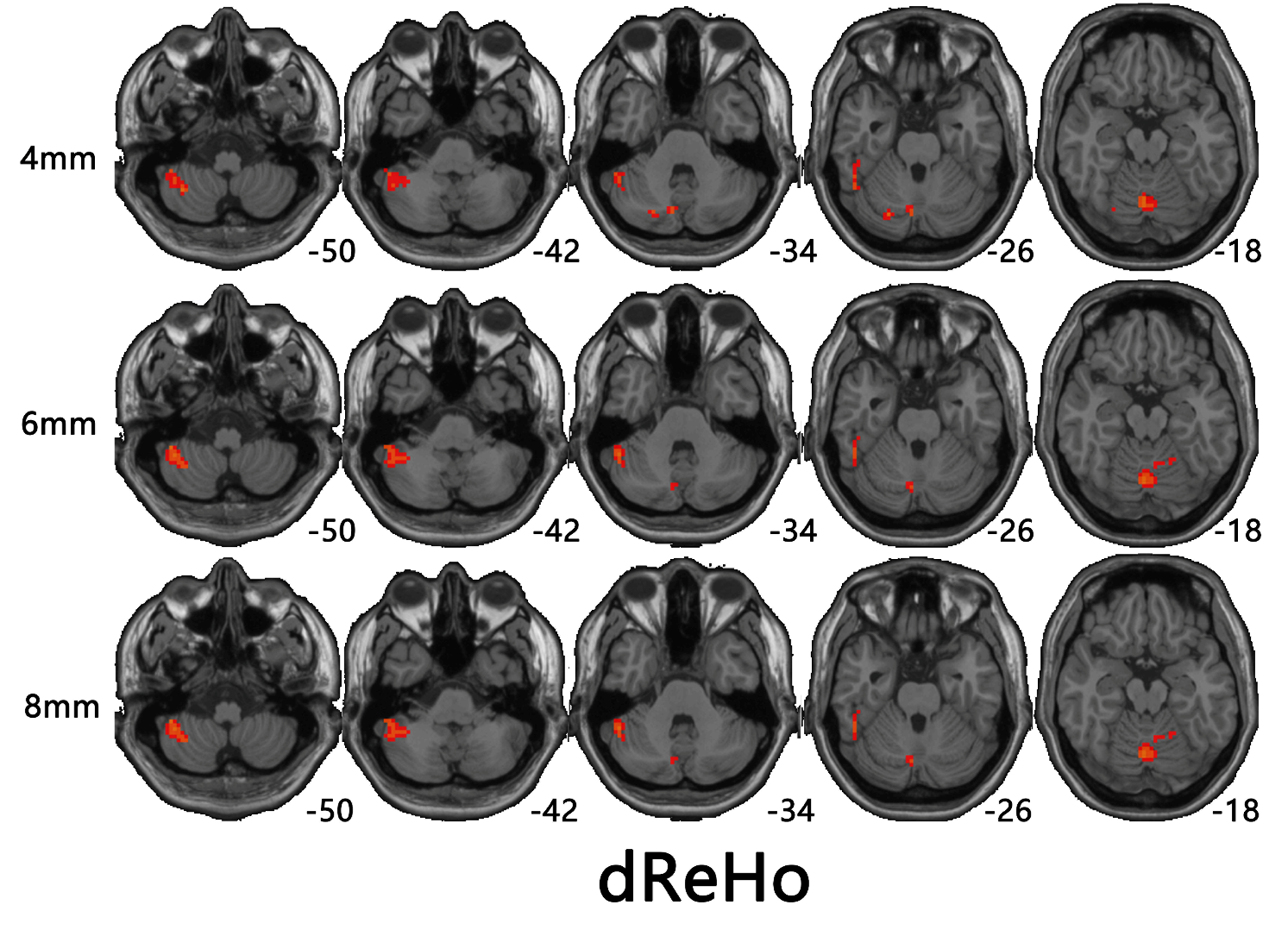


**Supplementary figure 4.** Differences in dReHo between the SCD, MCI, and NC groups with different Hamming window size. dReHo, dynamics of regional homogeneity; MCI, mild cognitive impairment; NC, normal control; SCD, subjective cognitive decline.

# Supplementary figure 5


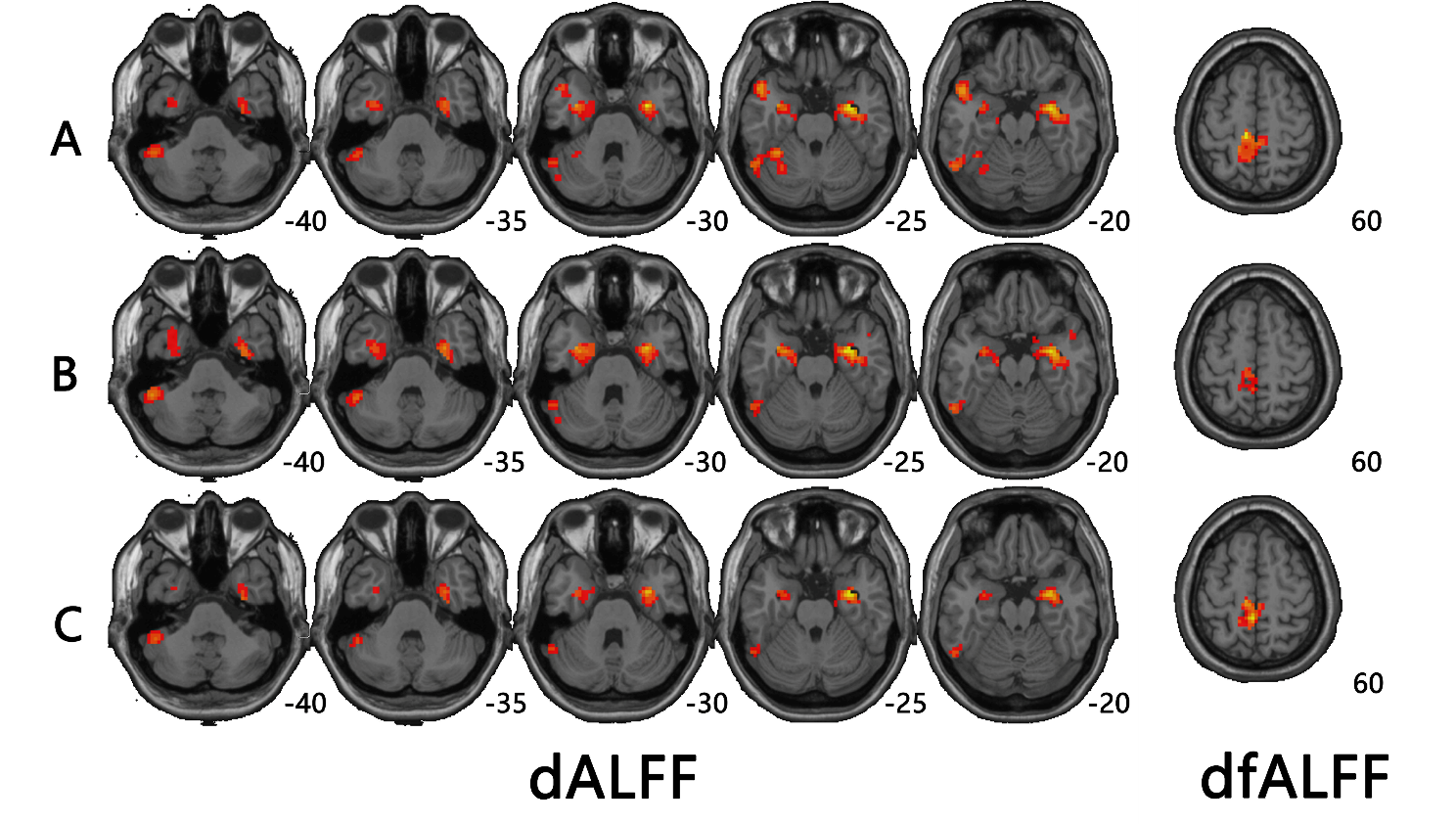


**Supplementary figure 5.** Differences in dALFF and dfALFF between the SCD, MCI, and NC groups with different nuisance signal regression. A, only white matter signal was regressed; B, both white matter and global signal was regressed; C, only global signal was regressed. dALFF, dynamics of amplitude of low-frequency fluctuations; dfALFF, dynamics of fractional amplitude of low-frequency fluctuations; MCI, mild cognitive impairment; NC, normal control; SCD, subjective cognitive decline.

# Supplementary figure 6


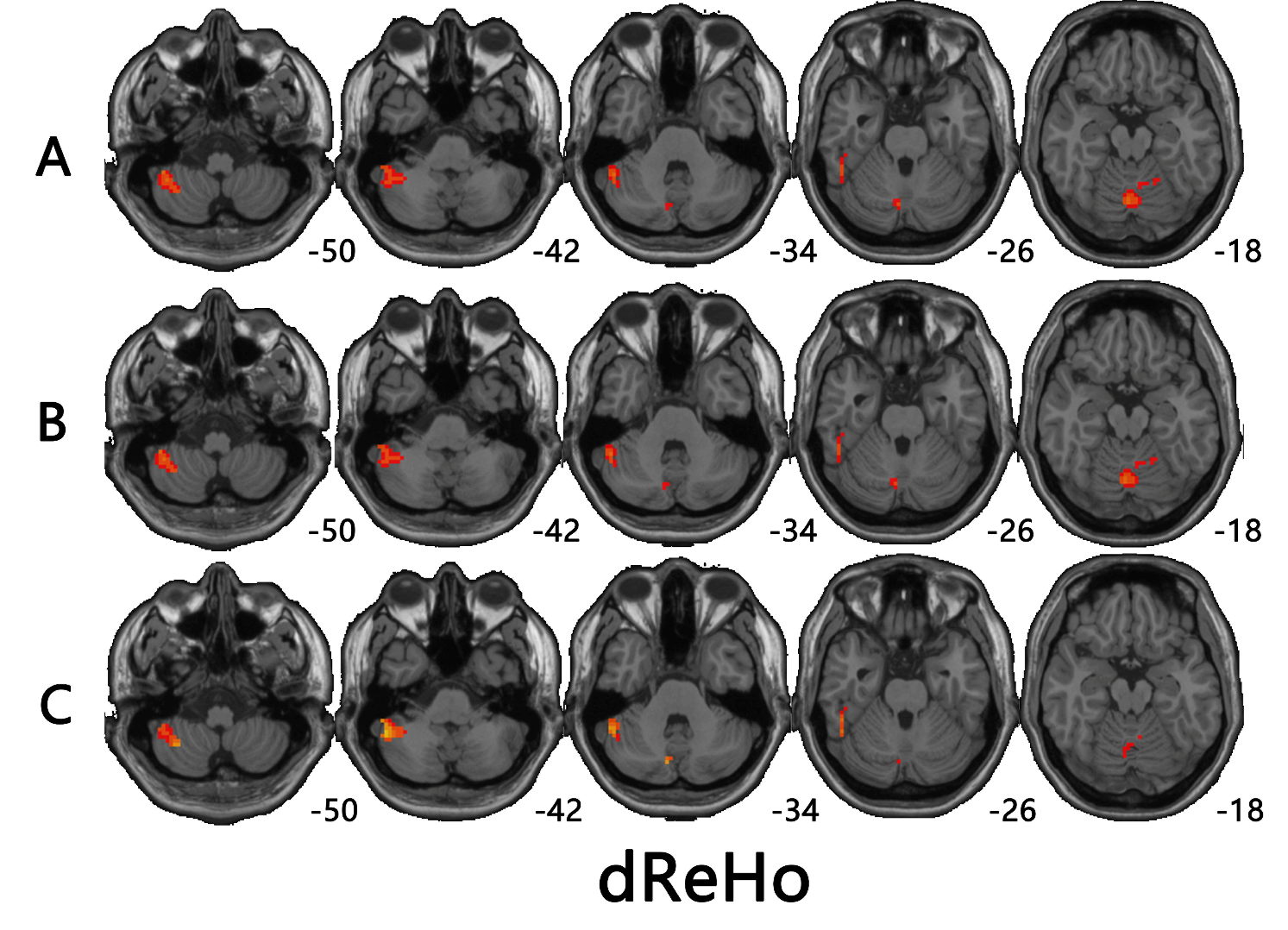


**Supplementary figure 6.** Differences in dReHo between the SCD, MCI, and NC groups with different nuisance signal regression. A, only white matter signal was regressed; B, both white matter and global signal was regressed; C, only global signal was regressed. dReHo, dynamics of regional homogeneity; MCI, mild cognitive impairment; NC, normal control; SCD, subjective cognitive decline.

# Supplementary figure 7


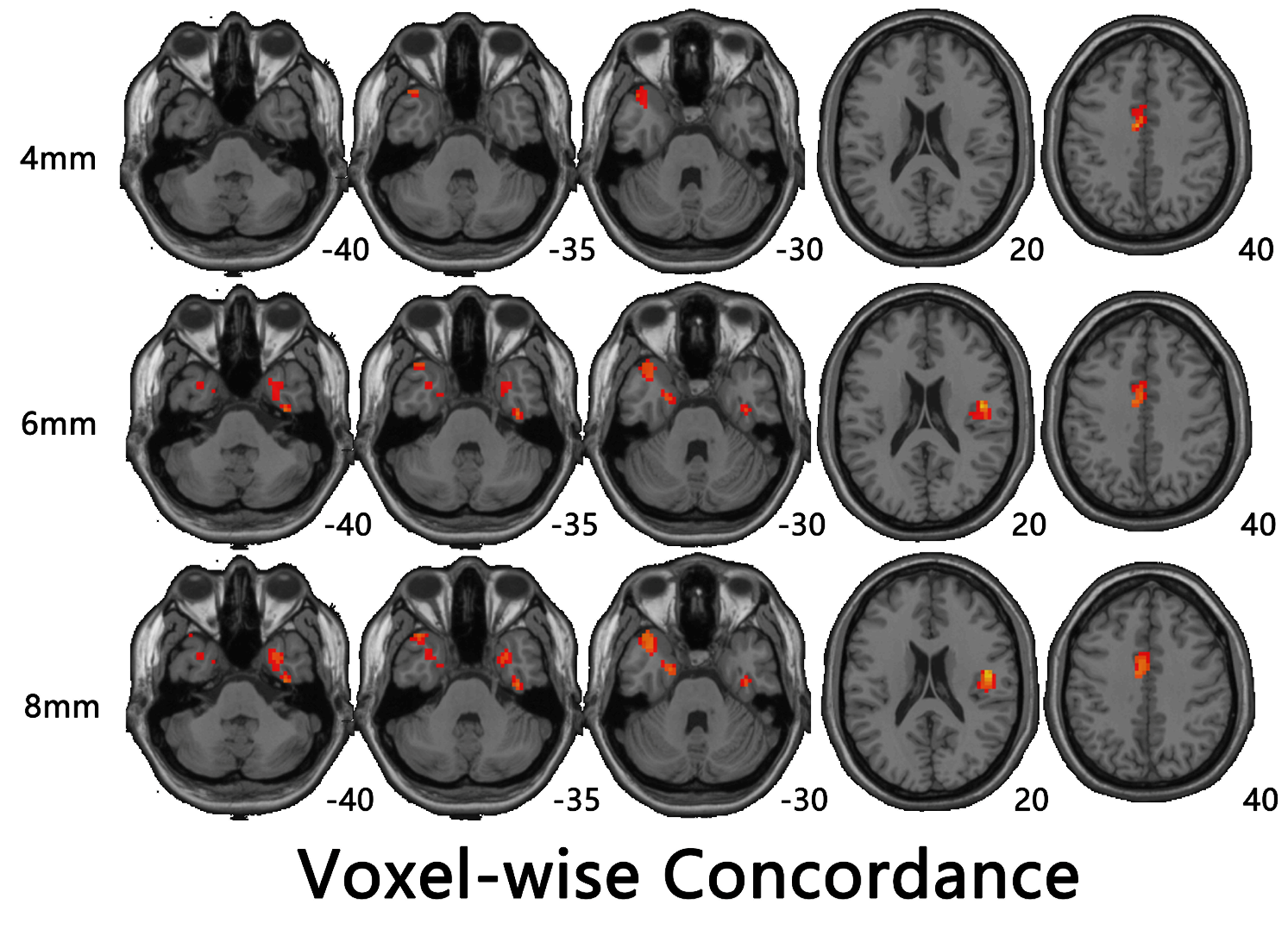


**Supplementary figure 7.** Differences in voxel-wise concordance between the SCD, MCI, and NC groups with different FWHM Gaussian kernel. FWHM, full width at half maximum; MCI, mild cognitive impairment; NC, normal control; SCD, subjective cognitive decline.

# Supplementary figure 8


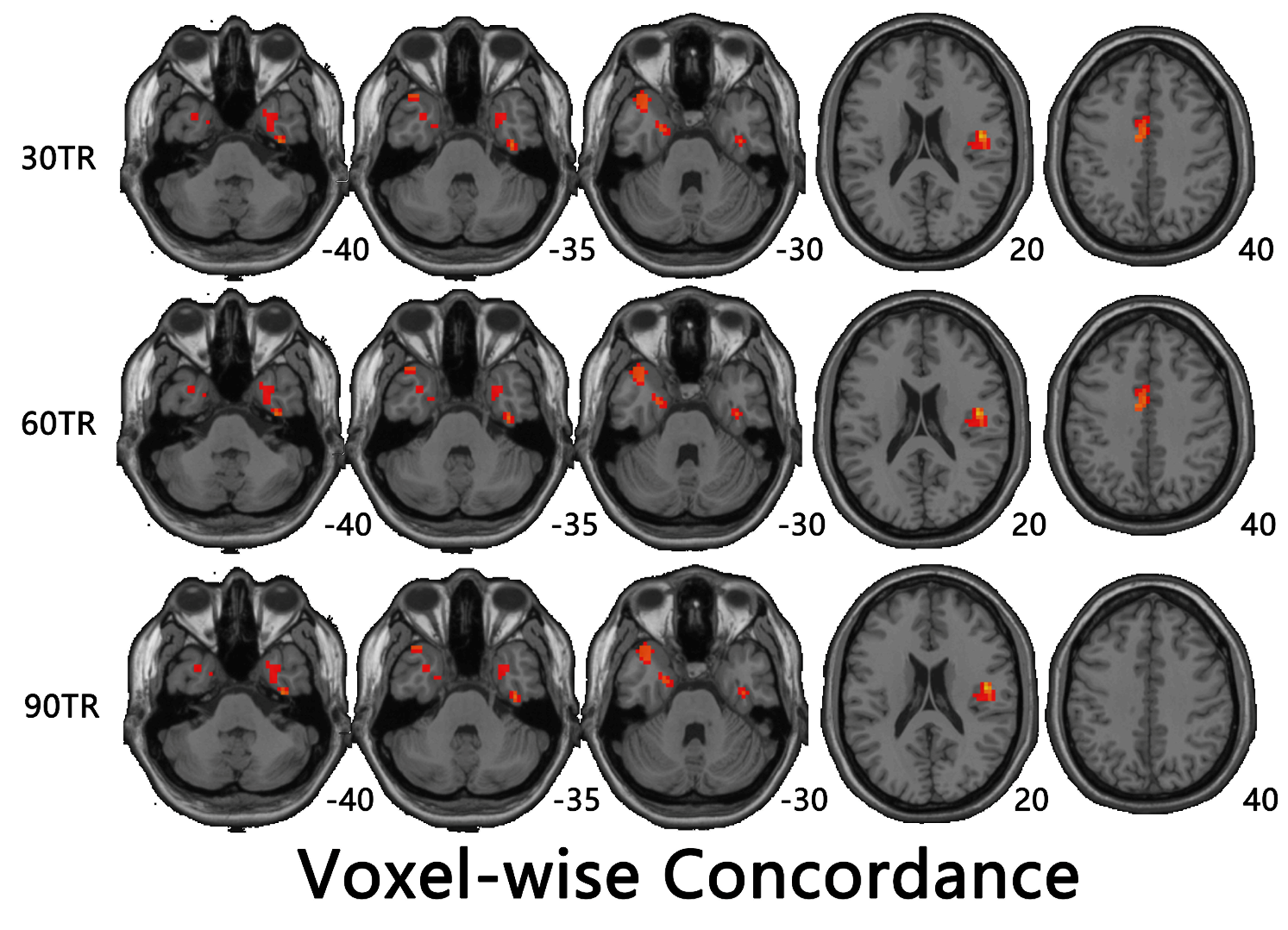


**Supplementary figure 8.** Differences in voxel-wise concordance between the SCD, MCI, and NC groups with different Hamming window size. MCI, mild cognitive impairment; NC, normal control; SCD, subjective cognitive decline.

# Supplementary figure 9


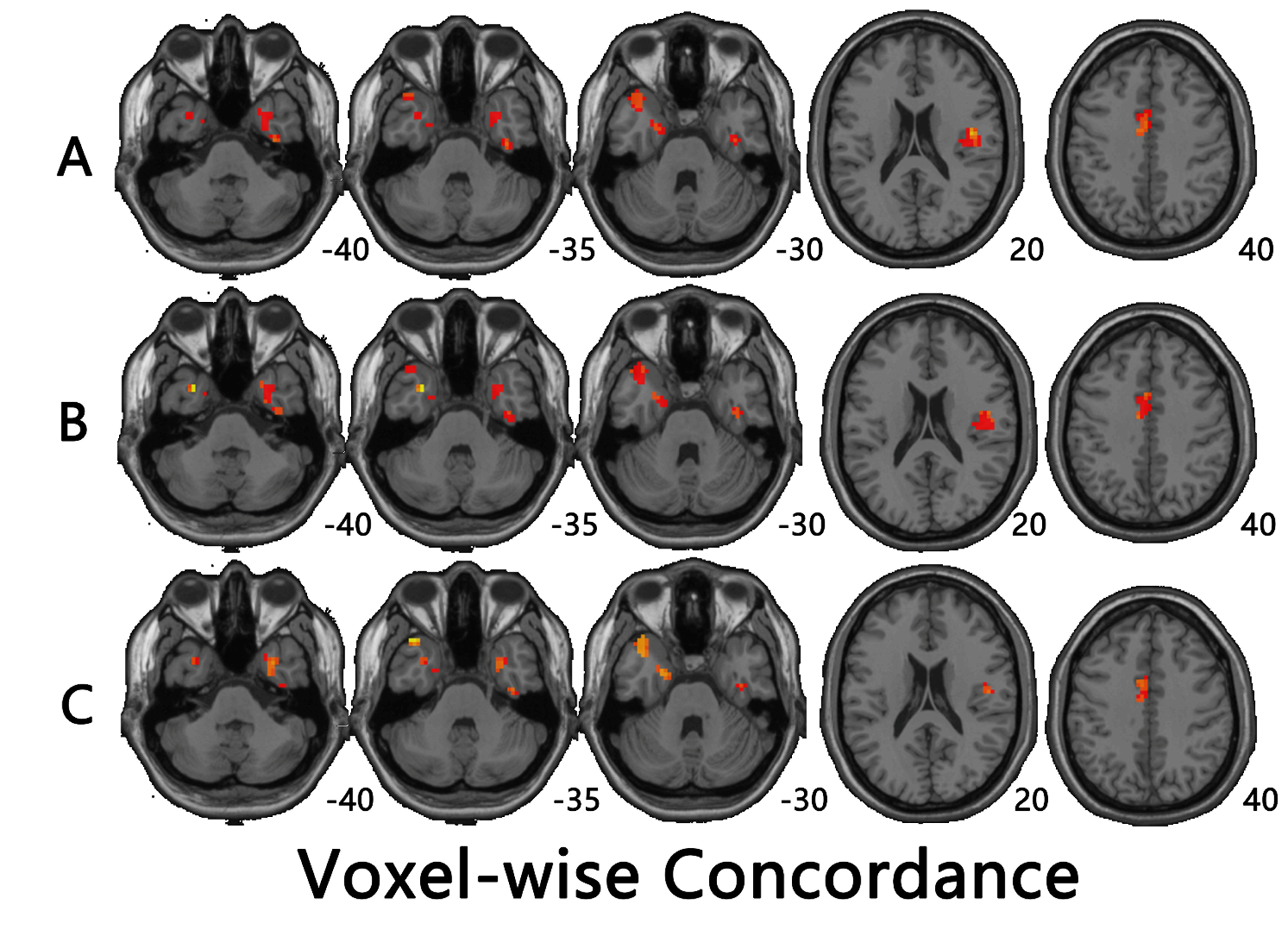


**Supplementary figure 9.** Differences in voxel-wise concordance between the SCD, MCI, and NC groups with different nuisance signal regression. A, only white matter signal was regressed; B, both white matter and global signal was regressed; C, only global signal was regressed. dReHo, dynamics of regional homogeneity; MCI, mild cognitive impairment; NC, normal control; SCD, subjective cognitive decline.
